# Differential responses of coral larvae to the colour of ambient light guide them to suitable settlement microhabitat

**DOI:** 10.1098/rsos.150358

**Published:** 2015-10-07

**Authors:** Marie E. Strader, Sarah W. Davies, Mikhail V. Matz

**Affiliations:** Department of Integrative Biology, The University of Texas at Austin, 1 University Station C0930, Austin, TX 78712, USA

**Keywords:** coral, settlement, light, exposure, green fluorescent protein, reef

## Abstract

Reef-building corals produce planktonic planula larvae that must select an appropriate habitat to settle and spend the rest of their life, a behaviour that plays a critical role in survival. Here, we report that larvae obtained from a deep-water population of *Pseudodiploria strigosa* settled more readily under blue light and in the dark, which aligns well with the light field characteristics of their natal habitat. By contrast, larvae of the shallow-water coral *Acropora millepora* settled in high proportions under blue and green light while settlement was less in the dark. *Acropora millepora* larvae also showed reduced settlement under red light, which should be abundant at shallow depth. Hypothesizing that this might be a mechanism preventing the larvae from settling on the exposed upwards-facing surfaces, we quantified *A. millepora* settlement in manipulated light chambers *in situ* on the reef. While *A. millepora* larvae naturally preferred settling on vertical rather than exposed horizontal surfaces, swapping the colours of upwards-facing and sideways-facing light fields was sufficient to invert this preference. We also tested if the variation in intrinsic red fluorescence in *A. millepora* larvae correlates with settlement rates, as has been suggested previously. We observed this correlation only in the absence of light, indicating that larval red fluorescent protein is probably not directly involved in light sensing. Our study reveals previously under-appreciated light-sensory capabilities in coral larvae, which could be an important axis of ecological differentiation between coral species and/or populations.

## 1. Introduction

The most critical behaviour for most sessile marine invertebrates exhibiting a bipartite life cycle is the selection of where to settle and metamorphose. Habitat selection by planktonic larvae strongly influences post-settlement survival [1,2], and much research has explored how settlement and metamorphosis are regulated in reef-building corals [3]. Selection of a suitable substrate can be influenced by cues associated with crustose coralline algae (CCA) [4,5], biofilms [6–8], substrate texture [9,10], substrate orientation [11] depth [1,6,12] temperature [13] and light [11,14,15].

Light has long been known to play a role in coral larval settlement. For example, larvae of several coral species (*Goniastrea aspera*, *Acropora tenuis*, *Oxypora lacera*) were shown to settle differently in response to variable light intensity, whereas larvae of *Goniastrea favulus* and *Montipora peltiformis* instead responded differentially to blue versus white light [11]. These settlement responses reflected light field’s characteristics of the depth ranges for these species [11]. In addition, variation in spectral sensitivity beyond blue-sensing has been suggested in the larvae of *Acropora palmata*, which preferentially settled on red fluorescent cable ties rather than on cable ties of other colours [16]. However, this observation cannot be easily interpreted in terms of larval ecology as light fields in this experiment were not measured or controlled. Another indication that larvae are sensitive to colours other than blue comes from an experiment in which reef-building coral larvae demonstrated electrophysiological sensitivity to pulses of red and blue–green light [17]. To date, however, direct demonstration of differential coral larval behaviours in response to intensity-controlled coloured light stimuli is lacking, and the potential ecological role of such photosensitivity remains unclear.

Coral larvae lack specialized visual structures, such as ocelli or a pigment ring, making it unlikely that they are able to perceive the direction of light (phototaxis) [18]. However, light-absorbing proteins ranging from blue-sensing cryptochromes [19] to long-wave sensitive opsins [20] have been identified in *Acropora* suggesting a potential mechanisms for light detection. In addition, by far the most abundant light-absorbing molecules in many coral larvae are green fluorescent protein (GPF)-like fluorescent proteins (FPs) [21]. Despite their high concentrations, biological functions of coral FPs remain controversial and may include proton transport [22,23] and photo-reduction of acceptor molecules [24], which could potentially initiate a light-sensing signalling cascade. In addition, it has been proposed that FPs play a role in photo-protection by depleting excess light energy present in its shallow-water habitat [25,26]. It is also conceivable that FPs might play a role in the light detection machinery as accessory pigments, modifying the spectral sensitivity of the primary sensory molecules such as opsins.

The objective of this study was to investigate whether behavioural responses of coral larvae to different light fields might extend beyond using light intensity or blue light as a depth cue, and evaluate whether variation, if present, in these light-sensory capacities might be attributable to differences in intrinsic fluorescence owing to FPs.

## 2. Methods

### 2.1 Pacific field season 1

In November 2011, at the Orpheus Island Research Station (OIRS), Great Barrier Reef (GBR), Australia, colonies of *Acropora millepora* were collected and maintained in flow-through raceways. Gametes were collected after spawning from two colonies, and larval rearing was performed as described in Meyer *et al.* [27] and in the electronic supplementary material, Methods. *Acropora millepora* larvae are aposymbiotic and remained so throughout culturing.

**Figure 1.**
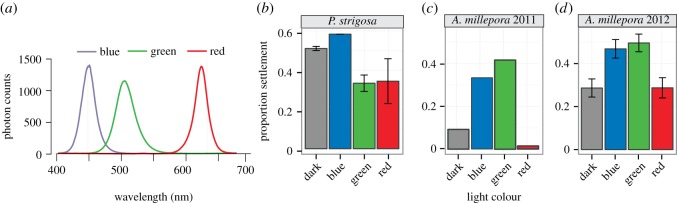
Larval settlement in three light treatments. (*a*) Light spectra of three light treatments (blue, green and red). (*b*) Proportion of settlement in *P. strigosa* at 48 h±s.e. (*c*) Proportion of settlement in *A. millepora* larvae from Pacific field season 1 at 12 h. (*d*) Proportion of settlement in *A. millepora* larvae from Pacific field season 2 at 8 h±s.e.

Three light treatments (red light, green light and blue light) were constructed with boxes uniformly lined with small LED lights on each of the four inner sides and upper surface with white paper lining the bottom surface. Incident light spectra for each treatment were collected using a USB2000 spectrometer (Ocean Optics, Dunedin, FL, USA) and corrected for the spectrometer’s spectral sensitivity. The total photon flux, estimated as the area under the spectral curve, was equalized across treatments by covering individual LED lights with aluminium foil (figure 1*a*). A darkness control was completely shielded from light using aluminium foil.

Individual larvae were placed in 96-well plates with approximately 200 μl of filtered sea water (FSW) and photographed (24 individuals per plate, one plate per treatment) using a fluorescent stereomicroscope MZ FL-III (Leica, Bannockburn, IL, USA) equipped with a double-bandpass F/R filter (Chroma no. 51004v2) and a Canon G6 camera. Larvae of *A. millepora* typically remain aposymbiotic until after metamorphosis [13]; therefore, the colours measured were not confounded by possible chlorophyll fluorescence. In addition, the filter used (Chroma no. 51004v2) detects red FP that is outside the range for cholorphyll fluorescence and therefore would not be detected even if it were present. After photographs, a small amount of finely ground CCA, a natural settlement inducer [4], was added to each well and plates were placed in light treatments (one light-exposed and one foil-wrapped plate per each light box). Individual larvae were scored for metamorphosis by the presence of septa after 24 h.

### 2.2 Caribbean field season 1

During the annual coral spawning event, August 2012, at the Flower Garden Banks National Marine Sanctuary (FGBNMS), gamete bundles were collected with mesh nets directly from three *Pseudodiploria strigosa* colonies and cross-fertilized for 1 h (see the electronic supplementary material, Methods for details). Larvae were transported to the University of Texas at Austin and reared as described in [5] for 5 days. *Pseudodiploria strigosa* larvae are aposymbiotic and remained so throughout culturing.

Individual larvae were transferred into approximately 200 μl FSW in 96-well plates and placed into the four light treatments (*n*=48 larvae per plate replicate, two plate replicates per treatment). A single drop of finely ground CCA slurry collected from the FGB [5] was added to each well. The coloured light treatments were set up as described in ‘Pacific field season 1’ (§2.1). The proportion of metamorphosed larvae was quantified after 48 and 72 h.

### 2.3 Pacific field season 2

In December 2012 at OIRS, gametes from two *A. millepora* colonies (designated A and B) were cross-fertilized reciprocally such that each colony served as either dam or sire, to generate two families of larvae (AB and BA).

Larval fluorescence per family was imaged as in Kenkel *et al.* [28] and described in the electronic supplementary material, Methods using 18–30 larvae per family. Red-green-blue values per larva were averaged within each family and a *t*-test (function *t*-test in R) determined the difference in redness between the families.

Settlement assays were performed on batches of 20 larvae in approximately 8 ml of FSW in six-well plates (three 6-well plates with three wells per larval culture per light treatment). The coloured light treatments were set up as described in ‘Pacific field season 1’ (§2.1). Proportions of metamorphosed larvae were quantified after 8 and 22 h (see the electronic supplementary material, Methods for details).

### 2.4 Pacific field season 3

In November 2013 at OIRS, gametes from six *A. millepora* colonies were cross-fertilized in bulk and reared as described in [27] and in the electronic supplementary material, Methods.

To manipulate light fields corresponding to horizontal and vertical orientations of the settlement surface, settlement chambers were constructed (see the electronic supplementary material, Methods and figure S2). All chambers contained an H-shaped assembly of small terracotta tiles soaked in ethanol extract of CCA (modified from Harrington *et al.* [2]), presenting a choice of settlement surfaces. Eight ‘coloured’ chambers were covered with theatrical gel filters to induce a redder light field at the vertical surfaces and a blue–green light field at the upward-facing horizontal surfaces; however, the light intensity remained much higher at the upward-facing horizontal surfaces (electronic supplementary material, figure S1). Eight ‘control’ chambers were wrapped in five sheets of diffusing white plastic to equalize the overall incident light intensity with the coloured chambers without affecting the spectral composition of the light field (electronic supplementary material, figure S2). The chambers were seeded with approximately 500 larvae (10 days post fertilization) and deployed on a near shore reef flat at 5 m depth for 36 h (two nights and one full day). The number of metamorphosed larvae on each tile surface was counted. This *in situ* experiment was conducted in the same local environment as the adults that were collected prior to spawning, which is consistent with the central temperature range for *A. millepora* in the GBR. Settlement chambers were deployed within very close proximity to each other, and control and treatment chambers were randomized. Therefore, differences in settlement between tile orientations are unlikely to be driven by temperature, as in the experiments by Winkler *et al.* [13] and Kenkel *et al.* [28].

**Figure 2.**
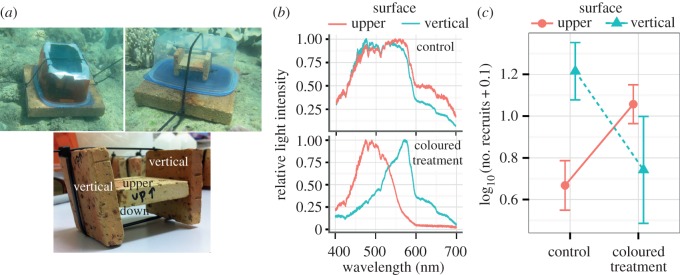
Larval settlement in *A. millepora* in manipulated light chambers *in situ* on the reef. (*a*) The experimental set-up for the coloured treatment chamber, control chamber (shown without white diffuser covering) and orientation of tiles. (*b*) Relative midday light spectra *in situ* at different surface exposures in control and coloured treatment chambers. *Y* -axis is relative light intensity, *x*-axis is wavelength in nanometres. (*c*) Effect of colour manipulation on settlement on upper and vertical substrate orientations. *Y* -axis is the log_10_-transformed recruit count (after adding a small value to zero count data points) ± s.e.

Ambient light spectra at the site and depth of deployed settlement chambers were measured using USB2000 spectrometer equipped with a 10 m long fibre optic cable (Ocean Optics) with the sensing tip of the fibre covered by diffusing Teflon tape, in four directions: facing up, facing down and facing to the side (towards shore and towards offshore) for both treatment and control chambers (figure 2*a*,*b* and electronic supplementary material, figure S1).

### 2.5 Statistical analysis

All statistical analyses were implemented in R, v. 3.1.2 (R Core Development Team, 2013). For Pacific field season 1, the function *fisher.test* was used to calculate the odds ratio of settlement between each light treatment (red, green and blue) compared with the darkness at the 24 h time point. Each larva was classified as being either red or green based on the fluorescent microphotographs and odds ratios of settlement between colour morphs in each treatment were calculated.

The remainder of the statistical analyses was performed using generalized linear-mixed (GLM) models (function *glmer*) defining the binomial response variable as counts of settled and not settled larvae. For Caribbean field season 1, we examined the effect of two fixed factors, time (48 and 72 h) and colour (blue, green, red and no light control) with random effect of plate. For Pacific field season 2, we examined three fixed factors, time (8 and 22 h), colour (blue, green, red and no light control) and family (AB and BA) with a random effect of well. For the settlement chamber experiment (Pacific field season 3), Poisson lognormal models were run with recruit counts as the response variable and fixed factors of tile surface exposure (up, down and vertical) and interaction with colour treatment (control or coloured). All GLM models were run allowing for over-dispersion (i.e. including a random effect of a data point). Significance of factors was determined using a likelihood ratio test (LRT). If a factor was found to be significant, we evaluated the significance of levels and interactions of each individual model. Modelling predictions were verified in R using the summary plots produced using the package *ggplot2* [29].

## 3. Results and discussion

Although the absorption of different wavelengths of light in water differs across aquatic systems, in general, ocean water strongly absorbs red light resulting in red wavelengths rapidly diminishing with depth (less than 5 m) [30,31]. Green light diminishes more gradually, with a significant decrease around 15 m [30,31], and blue light penetrates the deepest. Coral populations tested here live at very different depths, making it reasonable to hypothesize that these populations might detect differences in light spectra and use those cues to select a suitable microhabitat.

### 3.1 *Pseudodiploria strigosa*: light colour as depth cue

*Pseudodiploria strigosa* is a broadcast spawning coral common throughout the Caribbean, which is typically found at shallow depths [32]. However, this species is also abundant at the relatively deep-water Flower Garden Banks reef (more than 20 m) in the Gulf of Mexico [33]. Considering the light intensity and colour-dependent settlement in Mundy and Babcock [11], if larval settlement preferences match the parental habitat then *P. strigosa* larvae from this deep-water population are predicted to show diminished settlement under light conditions rich in long-wave green or red light, which are conditions typical for more shallow-water light environments. Our results show a trend towards this prediction: settlement was 1.5-fold lower under green and 1.4-fold lower under red light than in the dark (*p*=0.07 and 0.09, respectively), and 1.7-fold higher under blue light than in the dark (*p*=0.02; electronic supplementary material, table S2). The decrease in settlement under either green or red light relative to blue light was strongly statistically significant (*p*=4.0×10^−5^ and *p*=6.3×10^−5^ respectively; figure 1*b* and electronic supplementary material, table S2). This result demonstrates the ability of *P. strigosa* to respond to differences in light colour in a manner expectable for the deep-water population at the FGB. However, because this species normally exists in shallower water caution should be taken while extending this conclusion to the entire species. In fact, we can speculate that preference towards the blue light and avoidance of long-wave light could be unique to *P. strigosa* population from the FGB, being a result of local adaptation. Further studies involving profiling larval light responses across populations existing at different depths would be necessary to validate this hypothesis.

### 3.2 *Acropora millepora*: light colour as depth and surface exposure cue

*Acropora millepora* is found in shallow water, typically on reef flats, in lagoons and on upper reef slopes [32]. We predicted that this species would prefer settling in light environments rich in longer (green and red) wavelengths, which are abundant in shallow-water but diminish rapidly with depth [30,31]. In Pacific field season 1, larvae settled 4.8-fold better under blue and 6.8-fold better under green light compared with darkness (*p*_Fisher test_=2.1×10^−5^ and 3.1×10^−8^ respectively), and rarely settled under red light (settlement in red light was sixfold less than in darkness, *p*_Fisher test_=0.06; figure 1*c* and electronic supplementary material, table S1a). Replicating these experiments with modifications in the subsequent Pacific field season 2 again showed a significant effect of light treatment (*p*_LRT_=0.0003) with larvae settling 2.1-fold better under blue and 2.5-fold better under green light compared with darkness (*p*(>|*z*|)=0.002 and 0.0001, respectively). Once again, however, there was a significant decrease in settlement under red light compared with either blue or green (*p*(>|*z*|)=0.01 and 0.001 respectively; figure 1*d* and electronic supplementary material, table S2). These results, however, only show how larvae respond to a single light colour. Larvae in the water column are not exposed to one particular wavelength in isolation, but a mixture of multiple wavelengths ranging from ultraviolet to red. Therefore, it is possible that *A. millepora* larvae possess a more complex light detection mechanism than single-wavelength sensitivity, such as ratiometric detection of the proportion of green to red light in order to settle across species-specific depth range.

While further studies into light detection mechanisms in coral larvae are warranted, here we considered the possibility that light fields preferred by larvae reflected not only depth, but orientation (exposure) of the settlement surface. In the shallows, red wavelengths are more abundant on upwards-facing surfaces, whereas surfaces facing sideways into the phytoplankton-rich water column are predominantly illuminated by blue–green light (figure 2*b*). We therefore hypothesized that *A. millepora*’s apparent readiness to settle in green and blue light and reluctance to settle in red light would favour settlement in the shallows (where green light is still abundant) but on secluded vertical surfaces rather than on exposed horizontal surfaces. Coral larvae generally avoid settling on exposed upwards-facing surfaces [1,15,34], which is probably owing to avoidance of grazing, sedimentation, high levels of light stress in shallow water and/or competition with macroalgae during the earliest period of the juvenile coral’s life [1,10,35–37]. To test this hypothesis *A*. *millepora* settlement was quantified *in situ* under natural or manipulated light fields. In light-manipulated settlement chambers, the predominant light colours at the horizontal upwards-facing (exposed) and vertical sideways-facing (secluded) surfaces were flipped using light filters, such that the light field was redder at the secluded surface and blue–green at the exposed surface (figure 2*a*,*b*), whereas the overall light intensity remained much higher at the horizontal upward-facing surfaces (electronic supplementary material, figure S1). Control chambers were covered in diffuse white plastic instead of filters to compensate for the loss of the overall light intensity in the manipulated chambers (electronic supplementary material, figure S2). We found no effect of light treatment on overall settlement rate, a strong effect of tile orientation (*p*_LRT_=0.004) and a significant treatment by tile orientation interaction (*p*_LRT_=0.02). The majority of larvae settled on downward-facing horizontal surfaces in both the control and coloured treatment, a result that suggests light intensity plays a significant role in larval habitat selection, because downward-facing surfaces are generally more shaded (electronic supplementary material, figure S3). Notably, however, in the light-manipulated chambers, there was 2.4-fold more settlement on horizontal upper surfaces (Pr(>|*z*|)=0.04) and 2.2-fold less settlers on vertical surfaces (Pr(>|*z*|)=0.05), resulting in the inversion of the original relative preferences (figure 2*c*). The fact that the larval surface preferences can be inverted solely by manipulation of the spectral composition of the ambient light strongly suggests that light colour can serve as a cue for settlement not only within a species-specific depth range, but also in microhabitat-dependent manner.

### 3.3 Role of red fluorescence in light-sensing is not supported

**Figure 3.**
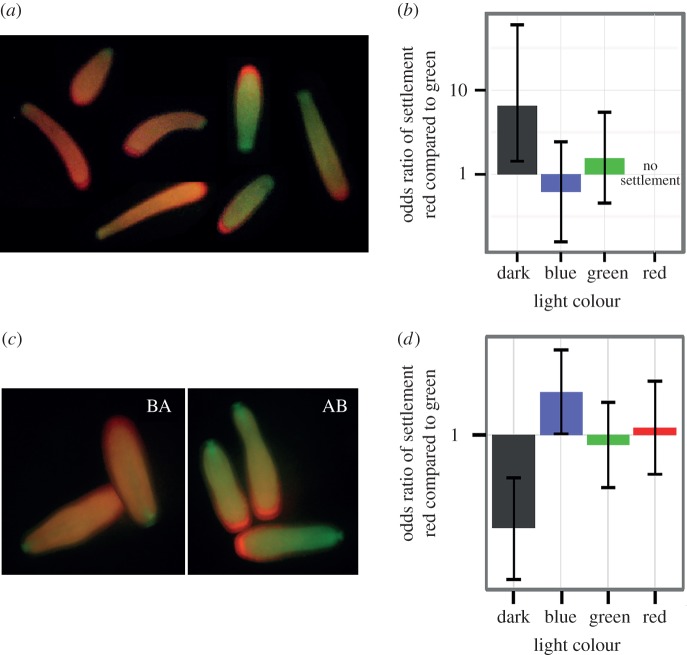
Larval settlement between colour morphs in different coloured light treatments. (*a*,*c*) Fluorescent polymorphism of full-sibling *A. millepora* larvae from 2011 (*a*) and 2012 (*c*). Larvae in (*a*) were generated from two parents and kept in the same culture. Larvae in (*c*) were generated from a reciprocal cross from two parents and were kept in separate cultures, cross AB (damA, sire B) and BA (damB, sire A). (*b*,*d*) Means and 95% credible intervals of log-odds ratios of settlement between red and green morphs in blue, green and red light treatments and in the dark control. (*b*) 2011, (*d*) 2012.

Most investigations into the role of FPs in corals have focused on the adult stage. Although both larvae and adults express a mixture of red and green phenotypes, in *A. millepora*, adult FPs are more similar to each other than the larval FPs of the same colour [25,38]. This suggests that gene copies of FPs probably play a different biological role in the larval and adult stages. Because FPs are hypothesized to play a role in the perception of light and larval colour morphs have shown differences in response to settlement cue [28], we tested whether colour-dependent settlement responses differed among fluorescent colour morphs. Because the excitation wavelengths for *A. millepora* red FP are within the green range [39], if red FP functioned as a light detection molecule then larvae with varying concentrations of red FP would show differential behaviour in the green light treatment. The two families of *A. millepora* from Pacific field season 2 were different in their average red fluorescence: the BA family was significantly redder than the AB family (*p*_*t*-test_=0.006, *t*_38_=−2.9; figure 3*c* and the electronic supplementary material, figure S4). The only significant difference between these families was the settlement rate in the dark: the redder family (BA) settled less than the greener family (AB; *p*_odds ratio_=0.0001; figure 3*d* and the electronic supplementary material, table S3). These results agree with observations in Kenkel *et al.* [28] showing that redder larval families settle less than greener ones; it is important to note that the settlement experiments in Kenkel *et al.* [28] were performed in the dark. However, because there was no difference in settlement between larval fluorescent morphs in any of the light treatments, the hypothesis that red and green FP play a direct role in light sensing during settlement is not supported. In adult corals, FP levels have been correlated with growth [26,40], photo-protection [25,26] and overall health [40–42]. In aposymbiotic larvae, it is possible that red FP contributes to photo-protection, allowing them to disperse in brightly sunlit surface waters. All these hypotheses implicate that FPs have diverse biological roles within a life stage and throughout the life cycle.

In the first experiment with *A. millepora* (Pacific field season 1), where individual larvae were monitored, it was possible to classify each larva as either a red or green morph to compare red versus green settlement rates (figure 3*a*). Once again, the most significant difference was observed in the dark (figure 3*b*; electronic supplementary material, table S1b), supporting the conclusion that neither red nor green fluorescence is directly associated with light sensing. Notably, this time red larvae settled significantly better than green larvae (*p*_Fisher test_=0.005). The contradiction with the other result as well as earlier results of Kenkel *et al.* [28], is most likely owing to the fact that in the present experiment the greener and redder larvae came from the same full-sibling family. It is possible that the factors responsible for within-family larval colour polymorphism do not affect the settlement behaviour in the same way as family-wide parental effects.

It should be noted that although our results do not support a direct role of red FPs in light sensing, they are insufficient to completely rule out the possibility of FP involvement in this function because they do not fully address the complexity of the larval FP complement. For instance, the complement of larval GFP-like proteins in *A. millepora* is substantially different from the adult counterpart and includes non/low-fluorescent green–yellow light-absorbing CP-like pigments which are derived from the red fluorescent amilFP597 found in adults [25,38]. Therefore, by characterizing our colour morphs by their emissions, we are not gaining a full representation of the absorption properties of the larvae. In addition, we did not consider the spatial distribution of FPs in the larvae. It is possible that multiple FPs and CPs, all with different absorption properties, work together along with other light-sensing proteins to detect light in a ratiometric way, and this could occur in spatially defined regions of the larvae such as the aboral-sensing region. To test these ideas, further work on the co-localization of FPs with known photoreceptors would be needed.

## 4. Conclusion

A previous study suggested that light intensity was the most important factor in larval settlement choice [15]. The data presented here add another layer of complexity and suggest that light colour may be an additional cue. We find that coral larvae may use coloured light cues not only as a depth gauge, as has been suggested previously [11], but also as an indicator of settlement surface orientation. It is notable that the two coral species studied here were substantially different in their settlement responses to coloured lights, although without additional experiments it would not be appropriate to generalize the observed preferences to each species as a whole. Previously, two other coral species (*A. palmata* and *Porites astreoides*) were observed to settle preferentially on red substrates [16], which indicates yet another type of response than we have observed here (both *A. millepora* and *P. strigosa* showed reduced settlement under red light). It can be generally concluded that modulation of coral larval settlement by the spectral composition of the ambient light can be complex and ecologically meaningful, ensuring favourable dispersion of larvae along the species-specific environmental range. In addition, these responses can be substantially variable among coral species and therefore can be an important mechanism of niche differentiation.

## Supplementary Material

One supplementary document contains three supplementary tables of statistical results, four supplementary figures and supplementary methods.

## Data accessibility

All R scripts and raw data are accessible in the Dryad digital repository: http://dx.doi.org/10.5061/dryad.1c4m9.
