# Supplementary material for: Differential responses of coral larvae to the colour of ambient light guide them to suitable settlement microhabitat
Source: R Soc Open Sci. 2015 Oct 7;2(10):150358. doi: 10.1098/rsos.150358 (PMC4632519; doi:10.1098/rsos.150358)
Supplement: One supplementary document contains three supplementary tables of statistical results, four supplementary figures and supplementary methods. [file rsos150358supp1.docx]

*Table S1*: *Acropora millepora* 2011 season. *(a)* Odds ratio of settlement between different light treatments and no light controls. *(b)* Odds ratio of settlement between red and green larval colour morphs in 4 light treatments (no larvae settled in red light treatment).

*(a)*

|  | | |
| --- | --- | --- |
| **Light Treatment Compared to Dark** | | |
| **Color treatment** | **Odds Ratio** | **P-value*** |
| Blue light | 4.8 🡹 | 2.1E-05 |
| Red light | 6.0 🡻 | 0.06 |
| Green light | 6.8 🡹 | 3.1E-08 |
|  |  |  |

* Fisher’s exact test

*(b)*

|  | | |
| --- | --- | --- |
| **Red larvae Compared to Green larvae** | | |
| **Light Treatment** | **Odds Ratio** | **P-value*** |
| Blue light | 1.6 🡻 | 0.54 |
| Green light | 1.5 🡹 | 0.6 |
| Darkness | 6.4 🡹 | 0.005 |
|  |  |  |

* Fisher’s exact test

*Table S2*: Statistics for *P. strigosa* and *A. millepora* (2012 and 2013 seasons).

| ***P. strigosa*** | | | |
| --- | --- | --- | --- |
| **Model** | | **AIC** | **Pr(>Chisq)** |
| glmer0: cbind(settled, not) ~ time + (1 \| index) + (1\|i2) | | 101.0 | < 0.0001 *** |
| glmer1: cbind(settled, not) ~ time + colour + (1 \| index) + (1\|i2) | | 93.5 | 0.004 ** |
| **Reference Condition** | **Condition** | **Pr(>\|z\|)** | **Fold-Difference** |
| colour (dark): |  |  |  |
|  | time (72 hrs) | 6.0e-10 *** | 2.6 🡹 |
|  | colour(blue) | 0.02 * | 1.7 🡹 |
|  | colour (green) | 0.07 . | 1.5 🡻 |
|  | colour (red) | 0.09 . | 1.4 🡻 |
| glmer1: cbind(settled, not) ~ time + colour + (1 \| index) + (1\|i2) | | 93.5 | 0.004 ** |
| **Reference Condition** | **Condition** | **Pr(>\|z\|)** | **Fold-Difference** |
| colour (blue): |  |  |  |
|  | time (72 hrs) | 6.0e-10 *** | 2.6 🡹 |
|  | colour(dark) | 0.02 * | 1.7 🡹 |
|  | colour (green) | 4.0e-05 *** | 2.4 🡻 |
|  | colour (red) | 6.3e-05 *** | 2.4 🡻 |
| ***A. millepora 2012*** | | | |
| **Model** | | **AIC** | **Pr(>Chisq)** |
| glmer0: cbind(settled, not) ~ time + (1 \| index) + (1\|i2) | | 727.4 | < 1e-15 *** |
| glmer1: cbind(settled, not) ~ time + colour + (1 \| index) + (1\|i2) | | 714.8 | 0.0003 *** |
| **Reference Condition** | **Condition** | **Pr(>\|z\|)** | **Fold-Difference** |
| colour (dark) |  |  |  |
|  | time (22 hrs) | < 2e-16 *** | 2.2 🡹 |
|  | colour (blue) | 0.002 ** | 2.1 🡹 |
|  | colour (red) | 0.5 | 1.2 🡹 |
|  | colour (green) | 0.0001 *** | 2.5 🡹 |
| **Reference Condition** |  |  |  |
| colour (red) |  |  |  |
|  | colour (blue) | 0.01 * | 1.8 🡹 |
|  | colour (green) | 0.001 ** | 2.2 🡹 |
| glmer2: cbind(settled, not) ~ time + colour + colour:cross + (1 \| index) + (1\|i2) | | 714.8 | 0.09 . |
| **Reference Condition** | **Condition** | **Pr(>\|z\|)** | **Fold-Difference** |
| colour (dark) & cross (ba) |  |  |  |
|  | colour (dark): cross (ab) | 0.02 * | 2.2 🡹 |
|  | colour (blue): cross (ab) | 0.2 | 1.6 🡻 |
|  | colour (green): cross (ab) | 0.5 | 1.3 🡹 |
|  | colour (red): cross (ab) | 0.8 | 1.1 🡹 |
|  |  |  |  |
| ***Settlement chambers experiment (A. millepora 2013)*** | | | |
| **Model** | | **AIC** | **Pr(>Chisq)** |
| glmer1: count ~ tileOrientation + (1 \| index) | | 172.0 | 0.004 ** |
| glmer2: count ~ tileOrientation + tileOrientation:treatment + (1 \| index) | | 168.0 | 0.02 * |
| **Reference Condition** | **Condition** | **Pr(>\|z\|)** | **Fold-Difference** |
| treatment (control): |  |  |  |
|  | tile orientation (up) | 0.003 ** | 3.5 🡻 |
|  | tile exposure (down): treatment (coloured) | 0.08 . | 2.0 🡹 |
|  | tile exposure (up): treatment (coloured) | 0.04 * | 2.4 🡹 |
|  | tile exposure (vertical): treatment (coloured) | 0.05 . | 2.2 🡻 |
| Signif. codes: 0 ‘***’ 0.001 ‘**’ 0.01 ‘*’ 0.05 ‘.’ 0.1 ‘ ’ 1 | | | |
|  |  |  |  |

*Table S3:* Odds ratios of settlement between BA and AB families under four light treatments

|  | | |
| --- | --- | --- |
| **Red larvae (BA) compared to green larvae (AB)** | | |
| **Colour treatment** | **Odds Ratio** | **P-value*** |
| Darkness | 2.7 🡻 | 0.0001 |
| Blue light | 1.6 🡹 | 0.04 |
| Red light | 1.0 🡹 | 0.8 |
| Green light | 1.1 🡻 | 0.6 |
|  |  |  |

* Fisher’s exact test

*FigureS1*: Non-normalized spectra in different directions for ambient light at depth for Pacific Field Season 3 (*a*), light within the coloured treatment (*b*) and light within the control treatment (*c*) ± SE. The curves represent light fields measured at different settlement surfaces (see legend) horizontal surfaces facing either up or down and vertical surfaces facing either the reef (“in”) or the expanse of sand patch (“out”). Numbers in parentheses are the number of times each measurement was taken. X-axis is wavelength in nanometers, Y-axis is photon counts.

*
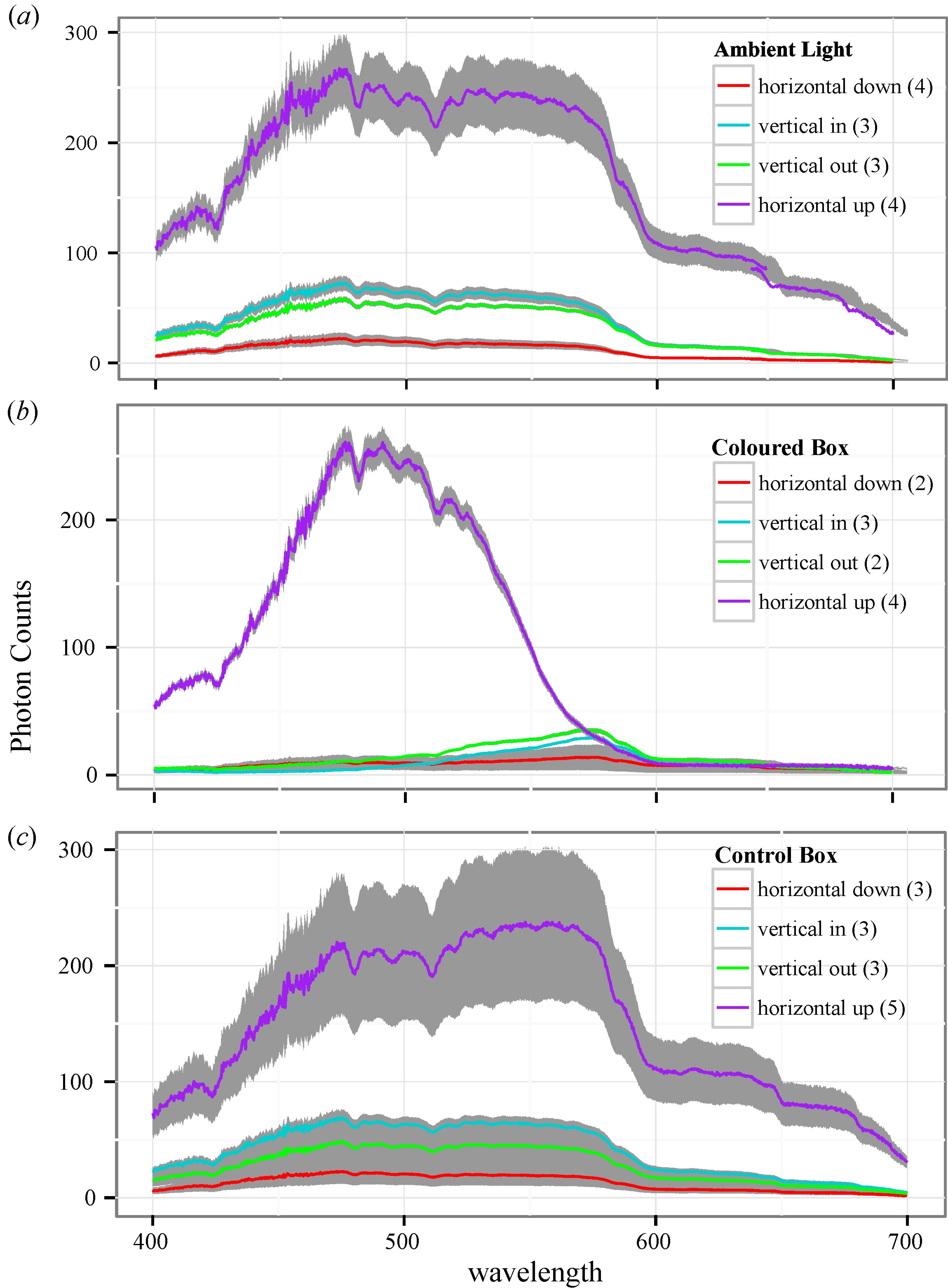
*

*FigureS2*: Equalized intensity between coloured and control chambers. (*a*) spectra with increasing layers of white, diffusing plastic. Y-axis is wavelength in nanometers (*b*) Number of diffusing layers necessary to equal total amount of light in coloured treatment (5).

*
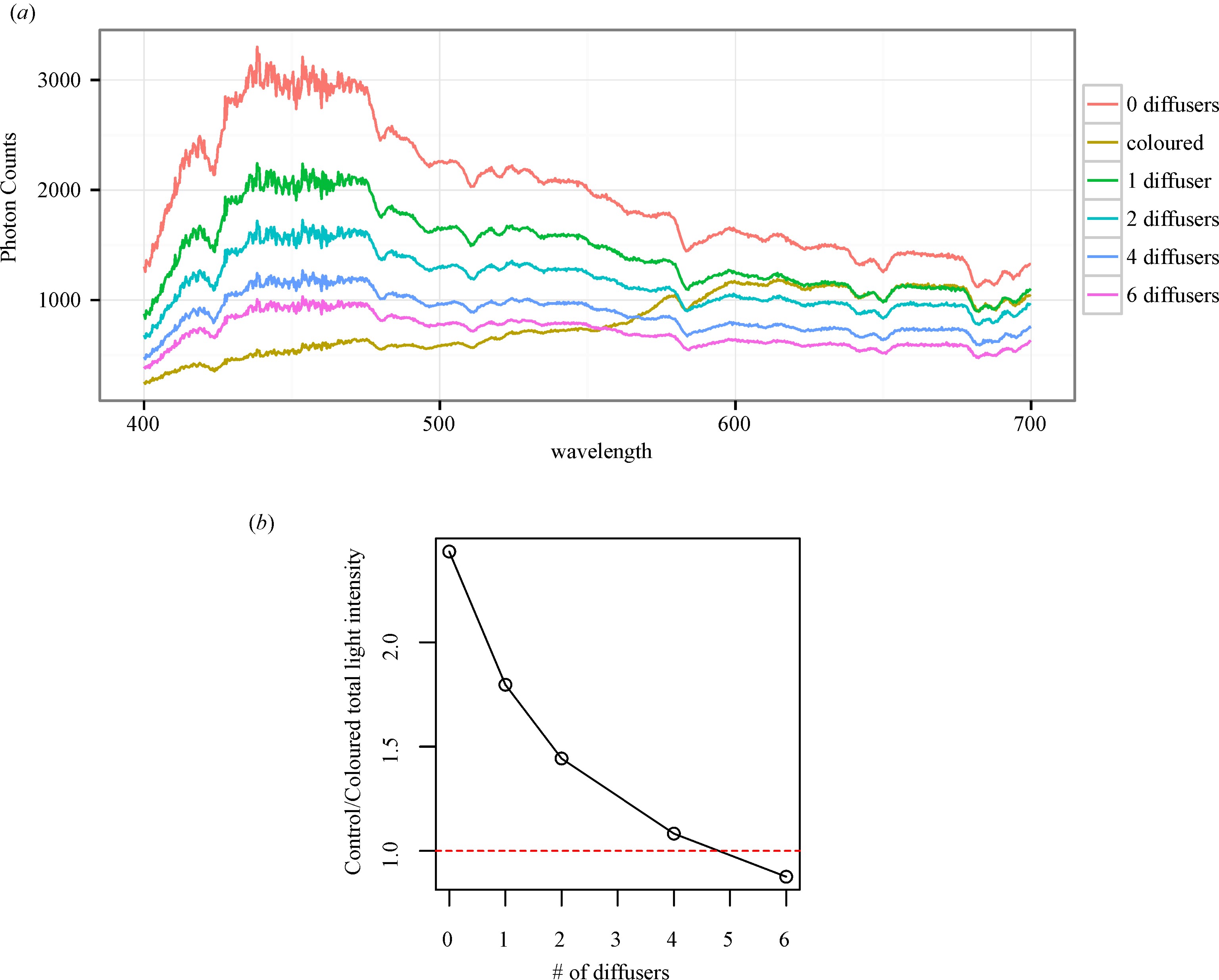
*

*FigureS3*: Total numbers of recruits of *A. millepora* in manipulated light chambers *in situ* on the reef on all surfaces (2013).

*Figure S4*: Differences in fluorescence in *A. millepora* larvae from 2012. Quantification of average fluorescence between crosses: average RGB values per larva per culture. The BA family has significantly more red larvae than the AB family (*P_t.test_*=0.006, t=-2.9, df=38).


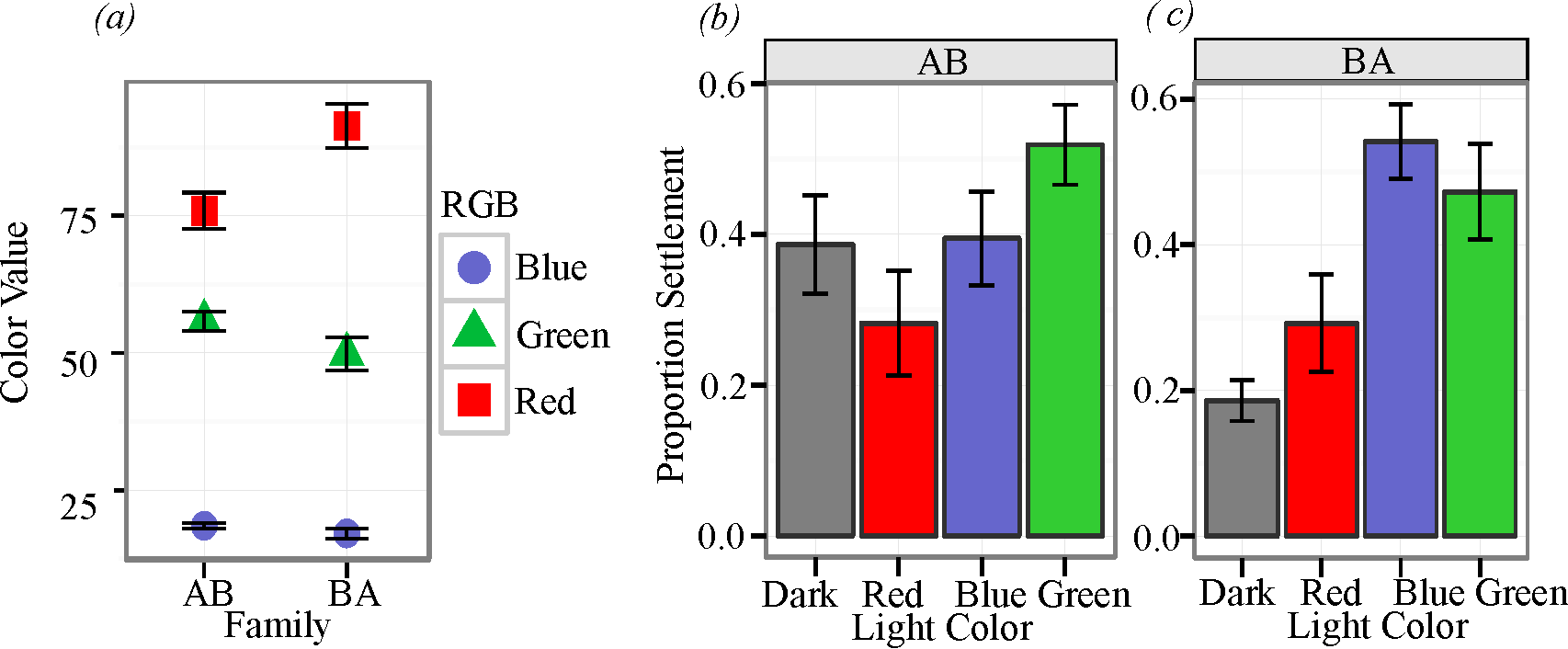


Supplementary Methods

Pacific Field season 1:

*A. millepora* colonies were collected from Little Pioneer Bay (18°36.989′S, 146°29.832′E). The corals were collected under the Great Barrier Reef Marine Park Authority permit # G10/33943.1. Approximately two hours prior to spawning time colonies were isolated in 20-gallon plastic bins. Colonies spawned on November 14, 2011 and gametes from 2 colonies were allowed to cross-fertilize for 2 hours. Excess sperm was washed out using gentle sieving and larval cultures were stocked in 0.5 mM filtered seawater (FSW) at the concentration of 1 larva per mL. Settlement assays began on the 9^th^ day post-fertilization.

Caribbean field season 1:

Samples were collected under the FGBNMS permit # FGBNMS-2012-002. Gametes were collected from 3 *Pseudodiploria strigosa* colonies (27.9200° N, 93.7100° W). Bundles were brought to the surface, cross-fertilized for one hour and excess sperm was removed by rinsing over 150 µm nylon mesh. Larvae were reared in 1 µm filtered seawater in three replicate plastic culture vessels at a density of 1 larva per 1mL. Larvae were transferred to the laboratory at the University of Texas at Austin in 50 mL conical tubes completely filled with FSW, with no air bubble remaining. Preliminary experiments determined that *P. strigosa* larvae reached competency 3-4 days post-fertilization. Settlement assays were performs on the 5th day post fertilization in light treatments constructed as described in Pacific Field Season 1 (red, green, blue light plus a dark control).

Pacific Field Season 2:

Samples for this work were collected under Great Barrier Reef Marine Park Authority permit number G10/33943.1. Replicate settlement experiments described in the previous sections were conducted with three light treatments (red, green and blue light plus a dark control) using locally collected CCA as settlement cues. Two adult *A. millepora* colonies (designated A and B) were used as parents to establish larval cultures as described above. Larvae from each cross were immobilized with ~0.04% paraformaldehyde and fluorescent photographs were taken immediately using a double-bandpass F/R filter (Chroma no. 51004v2). RGB values for each individual larva were calculated in ImageJ as the differences in RGB within the area of the larva normalized to the background. Settlement assays were performed 10 days post-fertilization. Larvae were scored for settlement beginning at 8 hours to account for spontaneous settlement that occurs in larvae that are older.

Pacific Field Season 3:

Following spawning, gametes from three Orpheus Island colonies and three northern colonies from Princess Charlotte Bay were cross-fertilized in bulk and reared at 1 larva per 2ml in eight culture replicates. Gentle aeration was provided from day 2 post-fertilization onward. The in situ settlement experiments were performed on larvae 13 days post fertilization. Light manipulation chambers were constructed using plastic rearing bins with upper surfaces lined with a blue filter (Rosco supergel filter #370) and side surfaces lined with a red/orange filter (Rosco supergel filter #20). Control chambers were covered with diffusing white plastic to equalize the overall light intensity between treatments (FigureS3).
